# Laparoscopic Resection of an Acute Rectosigmoid Intussusception Due to a Giant Pedunculated Lipoma

**DOI:** 10.7759/cureus.13798

**Published:** 2021-03-10

**Authors:** Jenny Sohn, Robert D Knox, Andrew Gilmore

**Affiliations:** 1 Department of Colorectal Surgery, Royal North Shore Hospital, Sydney, AUS; 2 School of Medicine, University of New South Wales, Sydney, AUS; 3 General Surgery, St George Hospital, Kogarah, AUS; 4 Department of General Surgery, Orange Base Hospital, Orange, AUS; 5 Department of Colorectal Surgery, Liverpool Hospital, Liverpool, AUS

**Keywords:** colonic intussusception, colonic lipoma, rectal bleeding, colorectal intussusception

## Abstract

Intussusception is the invagination and telescopic migration of a proximal segment of the gastrointestinal tract distally and is classically described in the paediatric age group, accounting for 95% of reported cases. Intussusception in adults is highly uncommon; its aetiology involves a wide spectrum of pathologies, namely, concerning malignancy and differing management pathways. In the management of adult intussusception, consideration must be given to the potential malignant nature of the lesion, as reducing a malignant segment could render dissemination. Intussusception with non-malignant lesions like lipomas has been described, usually involving the right and transverse colons. In this case report, we review a rare case of adult colorectal intussusception secondary to a submucosal sigmoid lipoma.

## Introduction

Intussusception in adults is highly unusual and involves a different spectrum of aetiologies as compared to the commonly described paediatric population. In the presentation of adult intussusception, malignancy is a primary concern. In the management of adult intussusception, consideration must be given to the potential malignant nature of the lesion, as reducing a malignant segment could render dissemination. Causes for non-malignant lesions like lipomas have been described in the literature and usually involve the right and transverse colon. In this case report, we review a rare case of a non-malignant cause of intussusception involving a submucosal lipoma of the sigmoid colon.

## Case presentation

A 46-year-old male presented to the emergency department (ED) with a two-day history of lower abdominal discomfort and passage of dark red blood per rectum, described as “jelly-like”. He described a sensation of prolapse, tenesmus and manual reduction of a palpable mass trans-anally the day prior to presentation. His past medical history included non-operative management of acute diverticulitis overseas, with colonoscopy four years prior that was reportedly unremarkable, and a traumatic right ocular injury. There was no family history of note.

**Figure 1:**
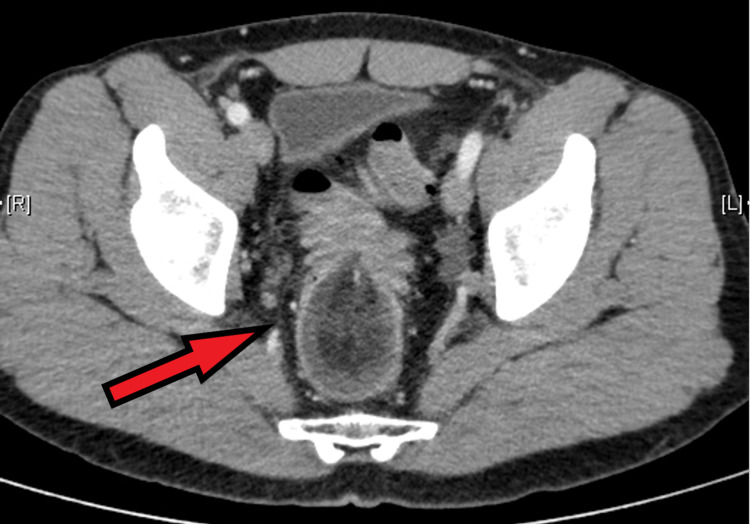
CT scan of the abdomen showing the colorectal intussusception

 The abdominal examination demonstrated a mildly tender lower abdominal mass extending below the pubic symphysis, with no significant distension. The digital rectal examination demonstrated a smooth mass occupying the mid rectum with fresh blood and mucus. Full blood count, c-reactive protein (CRP), and venous blood gas lactate levels were unremarkable. Computer tomography (CT) of the abdomen and pelvis revealed a long segment recto-sigmoid intussusception into the mid rectum (Figure 1).

The patient was consented for urgent flexible sigmoidoscopy with the possibility of proceeding to operative intervention trans-abdominally and was transferred to the operative theatre on the same day.

Sigmoidoscopy demonstrated a smooth, circumferential elongated mass arising from the proximal sigmoid, which was not reducible with insufflation or manual pressure. Due to its benign appearance, laparoscopy was performed and the polypoid lesion was successfully reduced into the distal sigmoid colon using atraumatic graspers. Formal laparoscopic anterior resection was then performed with trans-rectal stapled anastomosis, and the specimen was extracted through a transverse left lower quadrant incision.

**Figure 2:**
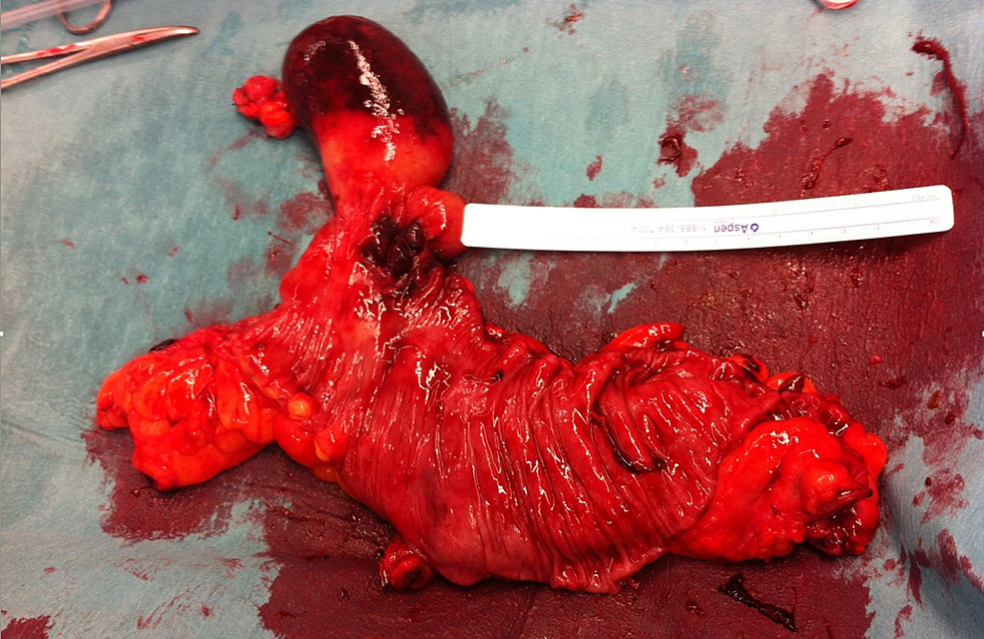
Operative specimen of giant lipoma in the sigmoid colon

Examination of the operative specimen revealed an apparent giant pedunculated lipoma (Figure 2). Histopathology confirmed a 55 x 45 x 40 mm pedunculated lipoma arising from the submucosa with overlying mucosal erosion, with several smaller submucosal lipomata adjacently. There were no atypical adipocytic features.

The patient made a swift and uneventful recovery and was discharged on Day 3 of admission.

## Discussion

Intussusception is the invagination and telescopic migration of a proximal segment of the gastrointestinal (GI) tract into the distal segment (intussuscipiens). The term is derived from the Latin “intus - within” and “sucipere - to take in”. Typically, this occurs in an anterograde direction, although retrograde intussusception has been reported in the context of gastrointestinal anastomoses. The first known presentation was in 1674 by Barbette, Amsterdam [1]. The classic presentation is in the paediatric age group, and intussusception in adults proposes an entirely different spectrum of pathologies and management. In the review of current literature, there were only three reported cases of sigmoid lipomas causing colo-rectal intussusception [2-4] as is with this case report.

As aforementioned, the paediatric age group accounts for 95% of reported intussusception cases and, conversely, it is an uncommon entity in adults [2]. In multiple small institutional review series [5-7], adult intussusception represents approximately 1% of all cases of intestinal obstruction and approximately one in 30,000 hospital admissions.

Causes of intussusception include disorders of peristalsis, structural or inflammatory lesions of the bowel wall. Luminal obstruction may progress to mesenteric involvement with vascular compromise followed by ischaemia and necrosis. Idiopathic causes account for 8%-20% of presented cases [5-7]. Metastatic melanoma deposits in the small bowel are described as the commonest single cause of adult intussusception [5].

Lipomas are benign tumours of adipose tissue with no premalignant potential. However, clinical differentiation between lipomas and well-differentiated liposarcomas are often difficult and their natural history is not well-described. In the GI tract, lipomas are the most common non-mucosal lesions but are uncommon tumours with a reported incidence of 0.15% on colonoscopic examinations and up to 4% on post-mortem examinations [8]. Lipomas can remain asymptomatic until approximately 3 cm to 3.5 cm in size and are termed giant once greater than 4 cm. They typically arise in the submucosal layer of the bowel wall, and endoscopically, they appear as smooth, rounded lesions with normal overlying mucosa. They may be suspected pre-operatively by CT appearance [9] (homogenous HU fat density) or maybe detected on T1-weighted MRI.

Ultrasound diagnosis and pneumatic reduction, the mainstay management of intussusception in children, are not indicated in adults given the common incidence of malignant aetiology. Investigation in adults largely relies on the use of CT or magnetic resonance imaging (MRI). X-ray imaging is not sensitive, and unlike in the paediatric age group, ultrasonography suffers from a similar lack of sensitivity. A series of 170 CT diagnoses of intussusception in adults reported that 93% of these cases were entero-enteric, and a length of the intussusception greater than 3.5 cm appeared to predict persistence and the need for surgery [9].

There is some debate in the literature as to whether an attempt at full or partial reduction prior to resection is more appropriate than a primary en-bloc resection of the intussuscepted segment. Consideration must be given to the potential malignant nature of the lesion, as reducing a malignant segment could render dissemination. Surgical resection has remained the mainstay method of management as previously published reports typically quote a malignant lead point in 50%-66% of cases of adult intussusception [5-7], which is likely to recur and progress without resection. However, in the largest published retrospective review of adult intussusceptions involving 1178 cases, the reported malignant aetiology was only 5.5% and 1.8% of large and small bowel intussusceptions, respectively [10]. This encourages further need to consider selective resections.

## Conclusions

Intussusception is an uncommon presentation in adults, especially in the anorectal canal. This case was unusual in presenting with the classic signs of intussusception, the presence of multiple lipomata, including one giant lipoma, and demonstrating that reduction can be safely undertaken to spare a patient the morbidity of a more extensive resection if a diagnosis of benign pathology is suggested. Recent retrospective data suggest that malignancy may not be as common a cause of intussusception in adults as originally thought, and there may be a greater role for pre-resection reduction or observation. Gastrointestinal lipomata should be considered for resection if greater than two centimetres to prevent the symptoms and complications of obstruction, ulceration, and uncommonly, intussusception.
